# Daidzein-Stimulated Increase in the Ciliary Beating Amplitude via an [Cl^−^]_i_ Decrease in Ciliated Human Nasal Epithelial Cells

**DOI:** 10.3390/ijms19123754

**Published:** 2018-11-26

**Authors:** Taka-aki Inui, Makoto Yasuda, Shigeru Hirano, Yukiko Ikeuchi, Haruka Kogiso, Toshio Inui, Yoshinori Marunaka, Takashi Nakahari

**Affiliations:** 1 Department of Molecular Cell Physiology, Graduate School of Medical Science, Kyoto Prefectural University of Medicine, Kyoto 602-8566, Japan; ikeuchi@koto.kpu-m.ac.jp (Y.I.); hkogiso@koto.kpu-m.ac.jp (H.K.); ymr18005@fc.ritsumei.ac.jp (Y.M.); 2 Department of Otolaryngology-Head and Neck Surgery, Graduate School of Medical Science, Kyoto Prefectural University of Medicine, Kyoto 602-8566, Japan; hirano@koto.kpu-m.ac.jp; 3 Research Center for Drug Discovery and Pharmaceutical Development Science, Research Organization of Science and Technology, BKC, Ritsumeikan University, Kusatsu 525-8577, Japan; t-inui@saisei-mirai.or.jp; 4 Saisei Mirai Clinics, Moriguchi 570-0012, Japan; 5 Research Institute for Clinical Physiology, Kyoto Industrial Health Association, Kyoto 604-8472, Japan

**Keywords:** nasal epithelia, cilia, intracellular Cl^−^ concentration, mucociliary clearance, amplitude of ciliary beating

## Abstract

The effects of the isoflavone daidzein on the ciliary beat distance (CBD, which is a parameter assessing the amplitude of ciliary beating) and the ciliary beat frequency (CBF) were examined in ciliated human nasal epithelial cells (cHNECs) in primary culture. Daidzein decreased [Cl^−^]_i_ and enhanced CBD in cHNECs. The CBD increase that was stimulated by daidzein was mimicked by Cl^−^-free NO_3_^−^ solution and bumetanide (an inhibitor of Na^+^/K^+^/2Cl^−^ cotransport), both of which decreased [Cl^−^]_i._ Moreover, the CBD increase was inhibited by 5-Nitro-2-(3-phenylpropylamino)benzoic acid (NPPB, a Cl^−^ channel blocker), which increased [Cl^−^]_i_. CBF was also decreased by NPPB. The rate of [Cl^−^]_i_ decrease evoked by Cl^−^-free NO_3_^−^ solution was enhanced by daidzein. These results suggest that daidzein activates Cl^−^ channels in cHNECs. Moreover, daidzein enhanced the microbead transport driven by beating cilia in the cell sheet of cHNECs, suggesting that an increase in CBD enhances ciliary transport. An [Cl^−^]_i_ decrease enhanced CBD, but not CBF, in cHNECs at 37 °C, although it enhanced both at 25 °C. Intracellular Cl^−^ affects both CBD and CBF in a temperature-dependent manner. In conclusion, daidzein, which activates Cl^−^ channels to decrease [Cl^−^]_i_, stimulated CBD increase in cHNECs at 37 °C. CBD is a crucial factor that can increase ciliary transport in the airways under physiological conditions.

## 1. Introduction

The nasal mucociliary clearance is maintained by the surface mucous layer and the beating cilia lining the nasal mucosa. Inhaled small particles, which are trapped by the surface mucous layer, are swept from the nasal cavity by the beating cilia. The impairment of beating cilia, such as primary ciliary dyskinesia, causes sinusitis [1,2,3]. Thus, beating cilia play a crucial role in the maintenance of a healthy nasal cavity. Substances that stimulate nasal ciliary beating are of particular importance to improve or prevent sinonasal disease, such as sinusitis.

We have shown that the activity of ciliary beating is maintained by two parameters, ciliary beat angle (CBA, an index of ciliary beat amplitude) and ciliary beat frequency (CBF) [4]. Many substances, such as procaterol, 3-isobutyl-1-methylxanthine (IBMX), ionomycin, cyclic adenosine monophosphate (cAMP), Ca^2+^, Sei-Hai-To (a Chinese traditional medicine), and carbocysteine, increase CBA and CBF in airway ciliary cells [4,5,6,7,8]. Moreover, we have reported that a decrease in intracellular Cl^−^ concentration ([Cl^−^]_i_) increases CBA, but not CBF [8]. Some agonists that stimulate Cl^−^ secretion, such as β_2_-agonists and carbocysteine, decrease [Cl^−^]_i_ by stimulating Cl^−^ release in many cell types [8,9,10,11]. Drugs that stimulate Cl^−^ secretion are used to stimulate mucociliary clearance in patients who have respiratory problems.

Isoflavones, which are known to be biological active compounds that are contained in soybeans and many other foods, are used as diet foods. Isoflavones, such as genistein, have been shown to stimulate Cl^−^ secretion in epithelial tissue via the activation of CFTR Cl^−^ channels [12,13,14]. Moreover, daidzein, an isoflavone, has been demonstrated to stimulate Cl^−^ channels in renal epithelial A6 cells [13]. These observations suggest that daidzein may stimulate Cl^−^ channels in ciliated human nasal epithelial cells (cHNECs), leading to an [Cl^−^]_i_ decrease, which increases the amplitude of ciliary beating, such as CBA. If so, daidzein may be an important substance for patients who have sinonasal problems.

Recently, we developed a method to measure CBD (ciliary beat distance, an index of ciliary beat amplitude) using a planner cell sheet of ciliated human nasal epithelial cells (cHNECs) in primary culture [8,15]. In this study, we examined the effects of daidzein on CBD and CBF in cHNECs. The goal of this study is to confirm that daidzein increases CBD in cHNECs through [Cl^−^]_i_ being decreased by the activation of Cl^−^ channels.

## 2. Results

The cells were first perfused with CO_2_/HCO_3_^−^-containing control solution for five min, and then with CO_2_/HCO_3_^−^-free control solution. All of the experiments were carried out under the CO_2_/HCO_3_^−^-free condition at 37 °C. The cHNECs, the CBFs of which were 8–14 Hz, were used for the experiments. The normalized CBD (CBD ratio) and CBF (CBF ratio) were used for comparing them among the experiments.

### 2.1. Video Images of cHNECs Activated by Daidzein

**Figure 1:**
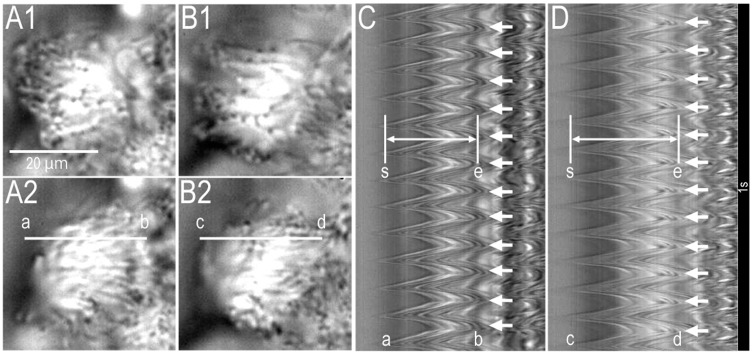
Video images of ciliated human nasal epithelial cells (cHNECs) in primary culture (apical view). The cHNECs were perfused with the CO_2_/HCO_3_^−^-free control solution. (**A**) Just before daidzein stimulation. (**B**) 15 min after daidzein stimulation (100 μM). Panels A1 and A2, and panels B1 and B2, show the start and the end of an effective stroke in a ciliary beating cycle, respectively. We set the lines “a–b’’ (A2) and “c–d’’ (B2) on the same beating cilia of a cHNEC in the video image to calculate the light intensity changes using the analysis program. (**C**) Changes in the light intensity of the line a–b (**A2**) in an unstimulated cHNEC. The image of light intensity changes enabled us to measure ciliary beat frequency (CBF) and ciliary beat distance (CBD). The two white lines in panel C show both the start (“s’’) and the end (“e’’) of an effective stroke. We measured the distance (pixels) from the line “s” to the line “e” as CBD. The number of arrows (right side) shows CBF. (**D**) Changes in the light intensity of the line “c–d’’ in the cHNEC 15 min after daidzein stimulation (100 μM). Daidzein stimulation enhanced CBD, but not CBF. Experiments were carried out at 37 °C.

Supplementary video S1A,B show a cHNEC (apical view) just before and 15 min after 100 µM of daidzein stimulation, respectively. Daidzein (100 µM) increases the amplitude of ciliary beating. Figure 1 shows the video images of a cHNEC (apical view) just before (panels A1 and A2) and 15 min after 100 µM of daidzein stimulation (panels B1 and B2), respectively. The beating cilia of cHNEC were observed from the apical side. Panels A1 and A2 or panels B1 and B2 show a cHNEC, the cilia of which are in the start position (A1 or B1) and the end position (A2 or B2) of an effective stroke, respectively. A line “a–b’’ was set on a beating cilium in the video image (panel A2). The analysis program reported changes in the light intensity on the line “a–b’’ (Figure 1C). Panel C shows the amplitude of ciliary beating as the distance between two lines (“s” and “e”) and CBF. We measured the distance between two lines (“s” and “e”) as CBD, and counted the number of beating waves as CBF. The values of CBD and CBF before daidzein stimulation were 84 pixels and 12 Hz, respectively. Next, the cHNECs were stimulated with 100 µM of daidzein. A line “c–d” in panel B2 was set on the same cilium as shown in panel A2. Changes in the light intensity of line “c–d” are shown in panel D. Panel D clearly shows that 100 µM of daidzein stimulation increased CBD, but not CBF. The values of CBD and CBF that were measured 15 min after 100 µM of daidzein stimulation were 112 pixels and 12 Hz, respectively. Thus, daidzein enhanced CBD in cHNECs.

### 2.2. Effects of Daidzein on CBD and CBF

**Figure 2:**
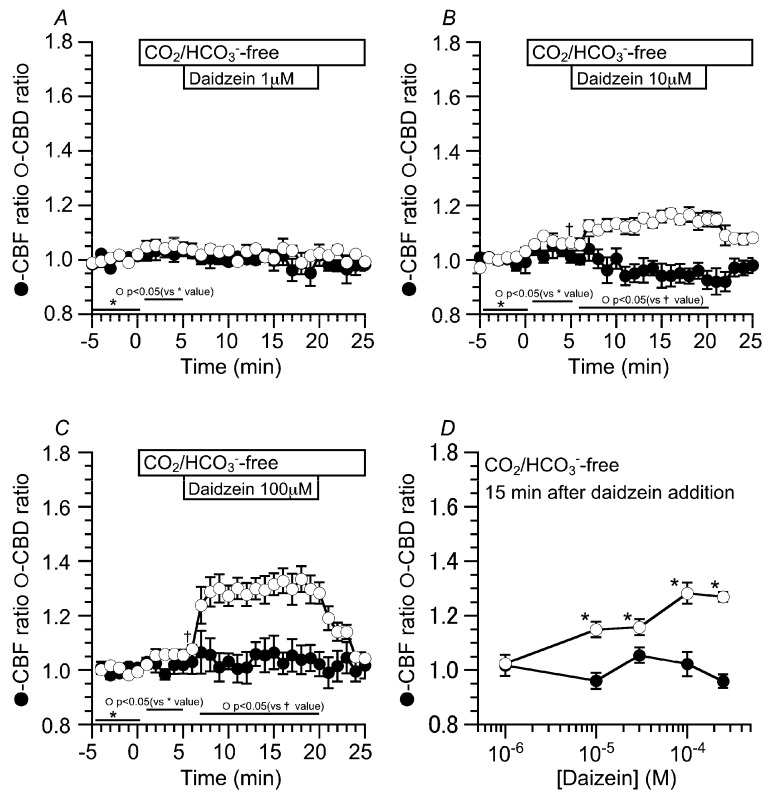
Concentration effects of daidzein on CBD and CBF in cHNECs at 37 °C. Prior to daidzein stimulation, cHNECs were perfused with the CO_2_/HCO_3_^−^-containing control solution, and then with the CO_2_/HCO_3_^−^-free control solution. The switch to the CO_2_/HCO_3_^−^-free control solution induced a small increase in CBD, but not CBF. (**A**) One µM of daidzein. Stimulation with one μM of daidzein did not change CBD or CBF. (**B**) 10 μM of daidzein. Stimulation with 10 μM of daidzein slightly increased CBD, but not CBF. (**C**) 100 μM of daidzein. Stimulation with 100 μM of daidzein increased CBD, but not CBF. (**D**) The concentration response study of daidzein. The ratio of CBD to CBF 15 min from the start of daidzein stimulation was plotted against the daidzein concentration. Daidzein increased CBD in a concentration-dependent manner without any increase in CBF. Daidzein at 100 µM maximally increased CBD in the cHNECs. * significantly different from the control value (*p* < 0.05).

The concentration effects of daidzein on CBD and CBF were examined. Prior to daidzein stimulation, the cHNECs were first perfused with the CO_2_/HCO_3_^−^-containing control solution for five min, and then with CO_2_/HCO_3_^−^-free control solution for further five min. The switch to the CO_2_/HCO_3_^−^-free control solution induced a small increase in CBD, but not CBF. The values of CBD ratio and CBF ratio five min after the switch were 1.02 ± 0.01 (*n* = 5) and 1.01 ± 0.03 (*n* = 5), respectively (Figure 2A). Stimulation with one µM of daidzein did not change CBD or CBF. Stimulation with 10 μM of daidzein increased CBD, but not CBF. The values of CBD and CBF 15 min after the switch were 1.14 ± 0.03 (*n* = 5) and 0.96 ± 0.03 (*n* = 5), respectively (Figure 2B). Stimulation with 100 μM of daidzein increased CBD, but not CBF. The values of CBD and CBF 15 min after the switch were 1.28 ± 0.04 (*n* = 6) and 1.02 ± 0.04 (*n* = 6), respectively (Figure 2C). The ratio of CBF to CBD 15 min after daidzein stimulation was plotted against the daidzein concentrations (Figure 2D). Daidzein increased CBD in a concentration-dependent manner without any change in CBF and maximally increased CBD at 100 μM (Figure 2D). In this study, the concentration of daidzein that was used was 100 μM throughout the experiments.

### 2.3. Effects of Daidzein on [Cl^−^]_i_, CBD, and CBF

**Figure 3:**
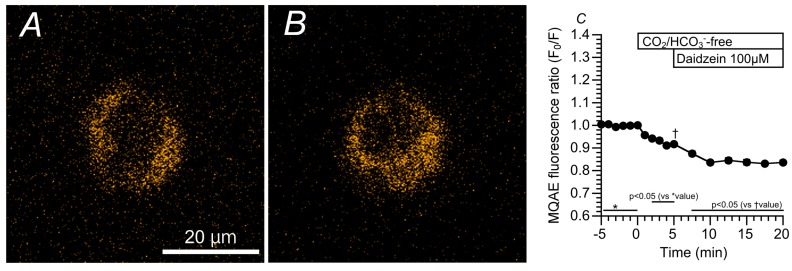
Changes in [Cl^−^]_i_ stimulated by daidzein. Prior to daidzein (100 µM) stimulation, cHNECs were first perfused with CO_2_/HCO_3_^−^-containing control solution for five min, and then with CO_2_/HCO_3_^−^-free control solution for a further five min. (**A**) The MQAE (*N*-ethoxycarbonylmethyl-6-methoxyquinolinium bromide, a Cl^−^ sensitive fluorescent dye) fluorescent image of a cHNEC just before daidzein stimulation. (**B**) The MQAE fluorescent image of a cHNEC 15 min after stimulation with daidzein (100 μM). The daidzein stimulation potentiated the intensity of MQAE fluorescence, indicating that daidzein stimulates a decrease in [Cl^−^]_i_. (**C**) Changes in the MQAE fluorescence ratio (F_0_/F). The switch to the CO_2_/HCO_3_^−^-free control solution decreased F_0_/F (F_0_/F five min after the switch = 0.92 ± 0.01, *n* = 6). Further stimulation with daidzein decreased F_0_/F (F_0_/F 10 min after the stimulation = 0.83 ± 0.01, *n* = 6).

We monitored changes in the [Cl^−^]_i_ of cHNECs using MQAE (*N*-ethoxycarbonylmethyl-6-methoxyquinolinium bromide, a Cl^−^ sensitive fluorescent dye) [11,16,17]. Daidzein has been reported to stimulate Cl^−^ secretion via the activation of the Cl^−^ channels [13], and the activation of Cl^−^ secretion appears to decrease [Cl^−^]_i_ [18]. MQAE fluorescent images of cHNECs are shown in Figure 3A (just before daidzein stimulation) and 3B (15 min after daidzein stimulation). Daidzein stimulation increased the intensity of MQAE fluorescence in the cHNECs, indicating that daidzein decreases [Cl^−^]_i_, as expected. Changes in the MQAE fluorescence ratio (F_0_/F) are shown in Figure 3C. The switch to the CO_2_/HCO_3_^−^-free control solution decreased F_0_/F (F_0_/F five min after the switch = 0.92 ± 0.01, *n* = 6). Further stimulation with daidzein decreased F_0_/F (F_0_/F 10 min after daidzein stimulation was 0.83 ± 0.01 (*n* = 6).

**Figure 4:**
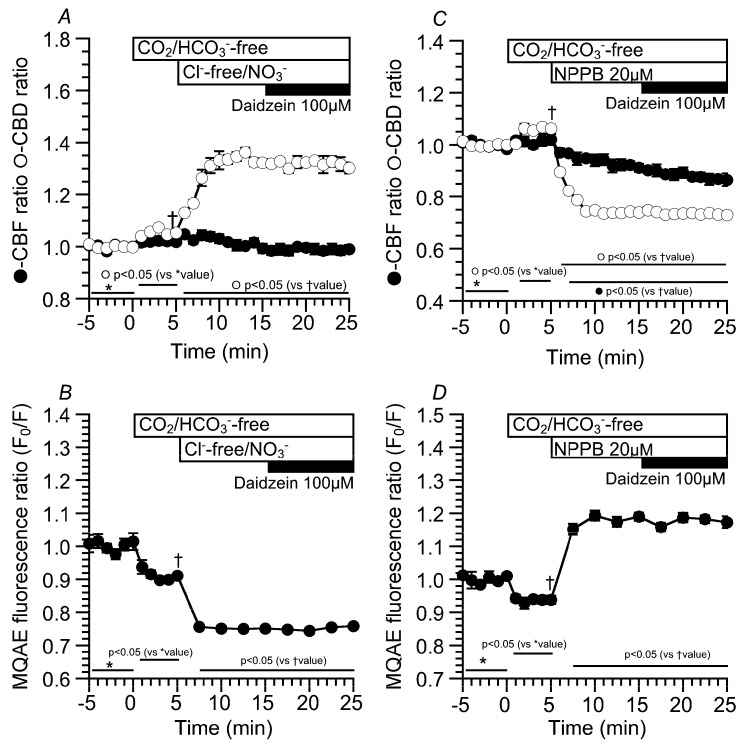
Effects of a low [Cl^−^]_i_ or a high [Cl^−^]_i_ on CBD and CBF in cHNECs. Experiments were carried out in the CO_2_/HCO_3_^−^-free control solution. To decrease the [Cl^−^]_i_ of cHNECs, a CO_2_/HCO_3_^−^-free Cl^−^-free NO_3_^−^ solution was used (**A**,**B**). In contrast, to increase [Cl^−^]_i_, NPPB was used (**C**,**D**). (**A**) The values of the CBD and CBF ratios just before the switch to the Cl^−^-free NO_3_^−^ solution were 1.05 ± 0.01 (*n* = 5) and 1.02 ± 0.01 (*n* = 8), respectively. The switch to the Cl^−^-free NO_3_^−^ solution immediately increased CBD, but not CBF. The values of CBD and CBF 10 min after the switch were 1.32 ± 0.01 (*n* = 5) and 0.99 ± 0.02 (*n* = 8), respectively. Further stimulation with 100 μM of daidzein did not change the CBD or CBF. (**B**) Decreases in the MQAE fluorescence ratio (F_0_/F) induced by the Cl^−^-free NO_3_^−^ solution. Switch to the CO_2_/HCO_3_^−^-free control solution decreased F_0_/F (F_0_/F five min after the switch = 0.91 ± 0.01, *n* = 7). Subsequent switch to the Cl^−^-free NO_3_^−^ solution decreased F_0_/F. The value of F_0_/F in the Cl^−^-free NO_3_^−^ solution 10 min after the switch was 0.75 ± 0.01 (*n* = 7). Further stimulation with 100 μM of daidzein did not change F_0_/F. (**C**) Decreases in CBD and CBF induced by NPPB. The values of CBD and CBF just before the addition of NPPB were 1.06 ± 0.01 (*n* = 5) and 1.02 ± 0.02 (*n* = 5), respectively. The addition of 20 μM of NPPB immediately decreased CBD and gradually decreased CBF. The values of CBD and CBF 10 min after NPPB addition were 0.74 ± 0.01 (*n* = 5) and 0.94 ± 0.02 (*n* = 5), respectively. The CBF decreased by NPPB alone reached a plateau within 20 min (CBF 20 min after NPPB addition = 0.88 ± 0.02 (*n* = 6, data not shown)). Further stimulation with 100 μM of daidzein did not affect CBD, but gradually decreased CBF. The values of CBD and CBF 10 min after daidzein stimulation were 0.73 ± 0.01 (*n* = 5) and 0.87 ± 0.02 (*n* = 5), respectively. (**D**) Increases in the MQAE fluorescence ratio (F_0_/F) induced by NPPB. The value of F_0_/F in the CO_2_/HCO_3_^−^-free control solution five min after the switch was 0.93 ± 0.01 (*n* = 7). The addition of 20 µM of NPPB increased F_0_/F (F_0_/F 10 min after NPPB addition = 1.19 ± 0.01, *n* = 7). Further stimulation with 100 μM of daidzein did not change F_0_/F (F_0_/F 10 min after daidzein stimulation = 1.17 ± 0.02, *n =*7).

The experiments were also carried out using CO_2_/HCO_3_^—^free, Cl^—^free, NO_3_^−^ solution to decrease [Cl^−^]_i_ [16,19]. The switch to the CO_2_/HCO_3_^−^-free control solution slightly increased CBD, but not CBF. The values of CBD and CBF five min after the switch were 1.05 ± 0.01 (*n* = 5) and 1.02 ± 0.01 (*n* = 8), respectively. The second switch to the CO_2_/HCO_3_^−^-free Cl^−^-free NO_3_^−^ solution immediately increased CBD, but not CBF. The values of CBD and CBF 10 min after the switch were 1.32 ± 0.01 (*n* = 5) and 0.99 ± 0.02 (*n* = 8), respectively. Further daidzein stimulation did not induce any increase in CBD or CBF (Figure 4A). Changes in the MQAE fluorescence ratio (F_0_/F) were also measured in the same protocol as an index of [Cl^−^]_I_ (Figure 4B). The switch to the CO_2_/HCO_3_*^−^*-free control solution decreased F_0_/F to 0.91 ± 0.01 (*n* = 7, five min after the switch), indicating a decrease in [Cl^−^]_i_. The second switch to the CO_2_/HCO_3_^—^free, Cl^—^free, NO_3_^−^ solution decreased F_0_/F (F_0_/F 10 min after the second switch = 0.75 ± 0.01, *n* = 7). Finally, daidzein stimulation did not induce any change in F_0_/F (Figure 4B). We also examined the effects of a high [Cl^−^]_i_ on CBD and CBF in cHNECs treated with the Cl^−^ channel blocker 5-Nitro-2-(3-phenylpropylamino) benzoic acid (NPPB) (20 µM), because NPPB has been shown to increase [Cl^−^]_i_ in airway ciliary cells [16]. Experiments were carried out in the absence of CO_2_/HCO_3_^−^. The switch to the CO_2_/HCO_3_^−^-free control solution slightly increased CBD, but not CBF, as shown in Figure 4A. The values of the CBD ratio and CBF ratio five min after the switch were 1.06 ± 0.01 (*n* = 5) and 1.02 ± 0.02 (*n* = 5), respectively. The addition of NPPB immediately decreased CBD and gradually decreased CBF. The values of CBD and CBF 10 min after the addition of NPPB were 0.74 ± 0.01 (*n* = 5) and 0.91 ± 0.01 (*n* = 5), respectively. Further daidzein stimulation did not change CBD and did not affect a gradual CBF decrease. The value of CBF reached a plateau within 25 min after NPPB addition. The values of CBD and CBF 10 min after daidzein stimulation were 0.73 ± 0.01 (*n* = 5) and 0.87 ± 0.02 (*n* = 5), respectively (Figure 4C). The MQAE fluorescence ratio (F_0_/F) was measured as an index of [Cl^−^]_i_. The switch to the CO_2_/HCO_3_^−^-free control solution decreased F_0_/F (F_0_/F five min after the switch = 0.93 ± 0.01, *n* = 7). The addition of NPPB increased F_0_/F to 1.19 ± 0.01 (*n* = 7, 10 min after NPPB addition), and further daidzein stimulation did not induce any change in F_0_/F (F_0_/F 10 min after the daidzein stimulation = 1.17 ± 0.02, *n* = 7) (Figure 4D). Thus, a decrease in [Cl^−^]_i_ increased the CBD. In contrast, an increase in [Cl^−^]_i_ decreased the CBD and CBF, although the actual values of [Cl^−^]_i_ were not measured.

**Figure 5:**
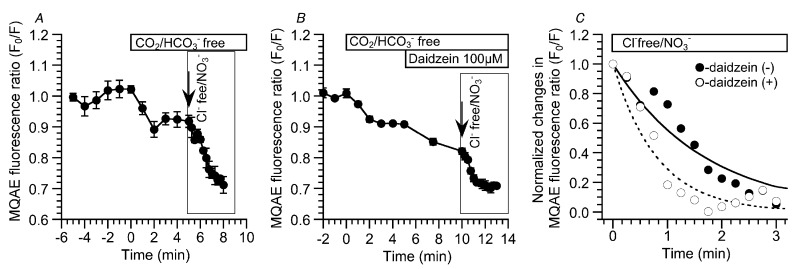
Enhancement of Cl^−^ efflux stimulated by daidzein. Experiments were carried out under the CO_2_/HCO_3_^−^-free condition. (**A**) Decreases in the MQAE fluorescence ratio (F_0_/F) induced by the Cl^−^-free NO_3_^−^ solution in the absence of daidzein. The value of F_0_/F just before the switch to the Cl^−^-free NO_3_^−^ solution was 0.91 ± 0.02 (*n* = 3). The switch to the Cl^−^-free NO_3_^−^ solution decreased F_0_/F (F_0_/F 2.5 min after the switch = 0.73 ± 0.02, *n* = 3). The value of F_0_/F plateaued within 2.5 min. (**B**) Decreases in the MQAE fluorescence ratio (F_0_/F) induced by the Cl^−^-free NO_3_^−^ solution in the presence of daidzein. The value of F_0_/F just before the addition of daidzein was 0.91 ± 0.01 (*n* = 3). Stimulation with 100 μM of daidzein further decreased F_0_/F (F_0_/F 5 min after the stimulation = 0.83 ± 0.01, *n* = 3). Switch to the Cl^−^-free NO_3_^−^ solution decreased F_0_/F (F_0_/F two min after the switch = 0.70 ± 0.02, *n* = 3). The value of F_0_/F reached a plateau within two min. (**C**) The time courses of F_0_/F decrease in the presence and the absence of daidzein. The normalized F_0_/Fs and fitted curves were plotted. The decreases in F_0_/F were fitted to an exponential curve, a·[exp(−t/τ)] (where a is the value at t = ∞ in each experiment, t is the time after procaterol stimulation, and τ is the time constant). Daidzein stimulation decreased τ from 1.70 ± 0.08 min (*n* = 3) to 0.81 ± 0.11 min (*n* = 3). Thus, daidzein stimulation accelerated the decreases in [Cl^−^]_i_ induced by the Cl^−^-free NO_3_^−^ solution, indicating that daidzein stimulates Cl^−^ efflux.

The results presented above suggest that daidzein stimulates Cl^−^ efflux via Cl^−^ channels. The decrease in the MQAE fluorescence ratio (F_0_/F) by a Cl^−^-free NO_3_^−^ solution was measured in the presence and absence of daidzein to confirm the daidzein-induced acceleration of [Cl^−^] decrease (Cl^−^ efflux) in cHNECs [17]. The experiments were carried out under the CO_2_/HCO_3_^−^-free condition. Switch to the CO_2_/HCO_3_^−^-free control solution decreased F_0_/F by approximately 10% within two min. The second switch to the CO_2_/HCO_3_^—^free, Cl^−^-free NO_3_^−^ solution decreased and plateaued F_0_/F within three min. The value of F_0_/F at three min after the application of the CO_2_/HCO_3_^—^free, Cl^−^-free NO_3_^−^ solution was 0.73 ± 0.02 (*n* = 3) (Figure 5A). Similar experiments were carried out during daidzein stimulation. In the CO_2_/HCO_3_^−^-free solution, daidzein stimulation decreased F_0_/F (F_0_/F five min after daidzein addition = 0.83 ± 0.01, *n* = 3). The switch to a CO_2_/HCO_3_^—^free, Cl^−^-free NO_3_^−^ solution decreased F_0_/F, which plateaued within two min (F_0_/F two min after the application of the CO_2_/HCO_3_^—^free, Cl^−^-free NO_3_^−^ solution = 0.70 ± 0.02, *n* = 3) (Figure 5B). To compare the effects of daidzein on Cl^−^ efflux, the time constants of the time courses in the [Cl^−^]_i_ decrease were calculated in the presence and absence of daidzein (boxes in Figure 5A,B). Decreases in the F_0_/F that were induced by the application of the CO_2_/HCO_3_^−^-free Cl^−^-free NO_3_^−^ solution were fitted to an exponential curve, a·[exp(−t/τ)], where a is the constant, t is the time after application of the CO_2_/HCO_3_^—^free, Cl^−^-free NO_3_^−^ stimulation, and τ is the time constant. The normalized values of F_0_/F and the fitted curves are shown in Figure 5C. The values of τ in the presence and absence of daidzein were 0.81 ± 0.11 min (*n* = 3) and 1.70 ± 0.08 min (*n* = 3), respectively. Thus, daidzein accelerated the [Cl^−^]_i_ decrease that was induced by the application of the CO_2_/HCO_3_^—^free, Cl^−^-free NO_3_^−^ solution, suggesting that daidzein stimulates Cl^−^ channels.

**Figure 6:**
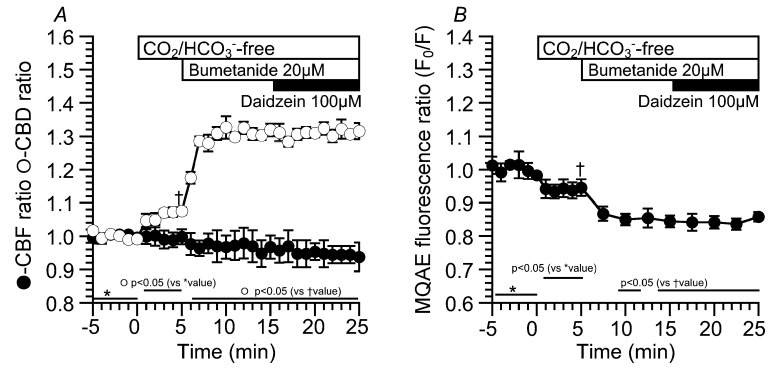
Effects of bumetanide on CBD, CBF, and [Cl^−^]_i_ in cHNECs. Experiments were carried out the CO_2_/HCO_3_^−^-free control solution. (**A**) Changes in CBD and CBF induced by bumetanide. The values of CBD and CBF just before the addition of bumetanide were 1.08 ± 0.01 (*n* = 5) and 1.00 ± 0.02 (*n* = 5), respectively. The addition of 20 μM of bumetanide immediately increased CBD, but did not increase CBF. The values of CBD and CBF 10 min after bumetanide addition were 1.32 ± 0.02 (*n* = 5) and 0.97 ± 0.03 (*n* = 5), respectively. Further stimulation with 100 μM of daidzein did not change CBD or CBF. The values of CBD and CBF 10 min after daidzein stimulation were 1.32 ± 0.02 (*n* = 5) and 0.94 ± 0.04 (*n* = 5), respectively. (**B**) Changes in the MQAE fluorescence ratio (F_0_/F). The value of F_0_/F just before the addition of bumetanide was 0.94 ± 0.03 (*n* = 4). The subsequent addition of 20 µM of bumetanide decreased F_0_/F (F_0_/F 10 min after bumetanide addition = 0.84 ± 0.02, *n* = 4). Further daidzein stimulation did not change F_0_/F.

Moreover, bumetanide (20 µM, an inhibitor of Na^+^/K^+^/2Cl^−^ cotransport (NKCC)) was used to decrease [Cl^−^]_i_ (Figure 6). Experiments were carried out under the CO_2_/HCO_3_^−^-free condition. Application of the CO_2_/HCO_3_^−^-free control solution increased CBD, but not CBF.

The values of CBD and CBF in the CO_2_/HCO_3_^−^-free control solution were 1.08 ± 0.01 (*n* = 5) and 1.00 ± 0.02 (*n* = 5). The addition of bumetanide increased CBD, but not CBF. The values of CBD and CBF 10 min after bumetanide addition were 1.32 ± 0.02 (*n* = 5) and 0.97 ± 0.03 (*n* = 5), respectively. Further stimulation with daidzein did not change CBD or CBF. The values of CBD and CBF 10 min after daidzein addition were 1.32 ± 0.02 (*n* = 5) and 0.94 ± 0.04 (*n* = 5), respectively (Figure 6A). The MQAE fluorescence ratio (F_0_/F) was also measured as an index of [Cl^−^]_i_ (Figure 6B). Switch to the CO_2_/HCO_3_^−^-free control solution decreased F_0_/F. Then, the addition of bumetanide decreased F_0_/F. The values of F_0_/F just before and 10 min after bumetanide addition were 0.94 ± 0.03 (*n* = 4) and 0.84 ± 0.02 (*n* = 4). Further daidzein stimulation did not change F_0_/F (F_0_/F 10 min after daidzein stimulation = 0.86 ± 0.01 (*n* = 4)). Bumetanide decreased F_0_/F, similar to the Cl^−^-free NO_3_^−^ solution under the CO_2_/HCO_3_^−^-free condition. Daidzein did not stimulate any increase in CBD or decrease in F_0_/F in an extremely low [Cl^−^]_i_, suggesting no driving force for Cl^−^ release via Cl^−^ channels.

### 2.4. Latex Microbeads Movement Driven by the Beating Cilia of cHNECs

**Figure 7:**
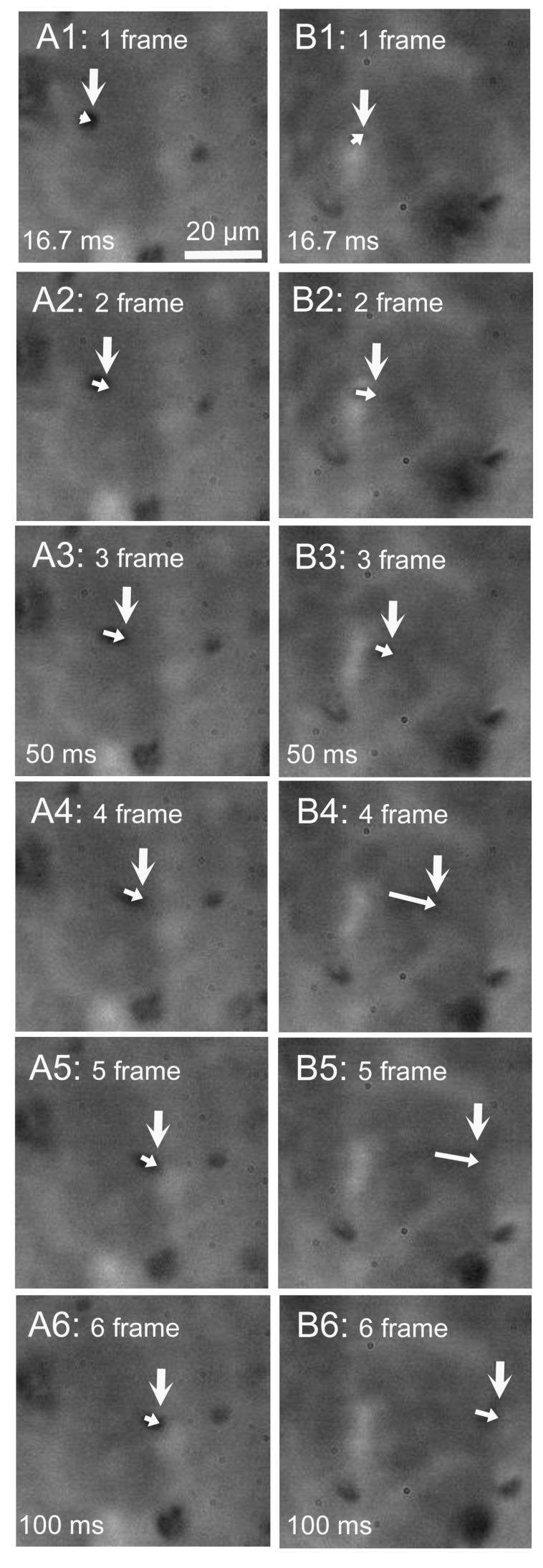
Movement of a latex microbead driven by beating cilia of a cHNECs. Experiments were carried out in the CO_2_/HCO_3_^−^-free solution. Movement of a microbead was recorded by a high-speed camera (60 fps). Panels A1–6 and B1–6 show six consecutive images taken every 16.7 ms. (**A**) Before daidzein stimulation. (**B**) Five min after daidzein stimulation. The initial position of the microbead is marked by a large white arrow in panels A1 and B1. Small white arrows in panels A1–6 and B1–6 show the movement of a latex microbead driven by the surface fluid flow for 16.7 ms. Stimulation with daidzein enhanced the microbead movement.

The beating cilia of cHNECs generate apical surface fluid flow. We observed the movement of latex microbeads, which was driven by the surface fluid flow. Latex microbeads (1 µm in diameter) were used for the experiments. A small amount of solution containing microbeads was added into the apical fluid, and movement of the microbeads was observed using a high-speed camera (60 fps). Microbeads that reached the surface of cHNECs were transported by the fluid flow generated by the beating cilia. Figure 7 shows six consecutive video frame images of cHNECs taken every 16.7 ms before (panels A1–6) and five min after daidzein stimulation (panels B1–6). The large vertical arrow in panels A1 and B1 show the position of a microbead that reached the apical surface of cHNECs. The large arrows in panels A2–6 and B2–6 show the position of microbeads every 16.7 ms. The small arrows in panels A1–6 and panels B1–6 show the distance that the microbead is moved by the surface fluid flow for 16.7 ms. In the case of cHNECs before and five min after daidzein stimulation, microbeads were, respectively, transported by approximately 20 µm (panels A1–6) and 33 µm over a period of 100 ms (panels B1–6). Thus, daidzein stimulation enhanced the microbead movements driven by the surface fluid flow. Supplementary Materials S2A,B show the videos of the microbead movement before and five min after daidzein stimulation, respectively.

**Figure 8:**
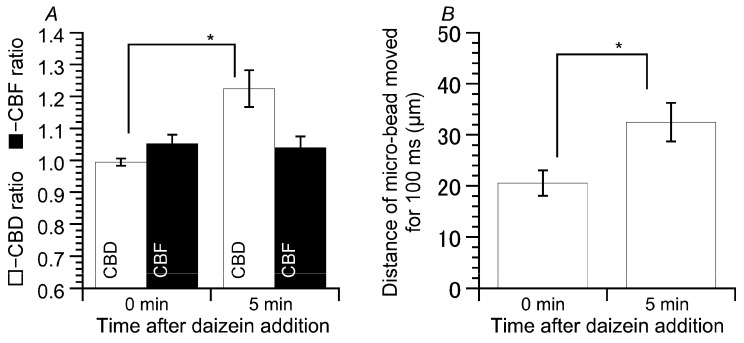
Effects of daidzein on microbead movements. (**A**) Changes in CBD and CBF before and five min after daidzein stimulation. The cHNECs were first perfused with the CO_2_/HCO_3_^−^-containing control solution for five min, and then with the CO_2_/HCO_3_^−^-free control solution for further five min. Stimulation with daidzein increased CBD, but not CBF. (**B**) Enhancement of microbead movement by daidzein stimulation. Daidzein stimulation enhanced the microbead movement. * significantly different (*p* < 0.05).

We measured CBF, CBD, and microbead movement using cHNEC cell sheets (Figure 8). Daidzein stimulation increased CBD, but not CBF (Figure 8A). The values of CBD and CBF before the stimulation were 0.99 ± 0.01 (*n* = 6) and 1.05 ± 0.03 (*n* = 6), and those five min after the stimulation were 1.22 ± 0.06 (*n* = 6) and 1.04 ± 0.03 (*n* = 6), respectively. The distances that the microbeads were moved by the surface fluid flow were measured before and five min after daidzein stimulation. Daidzein stimulation enhanced the distance that the microbead moved over a period of 100 ms from 20.5 ± 2.5 µm (*n* = 6, before the stimulation) to 32.5 µm ± 3.8 (*n* = 6, five min after daidzein stimulation) (Figure 8B). Thus, an increase in CBD stimulated by daidzein enhanced the microbead movement.

### 2.5. Effects of Ca^2+^ and cAMP on CBD and CBF Stimulated by Daidzein

**Figure 9:**
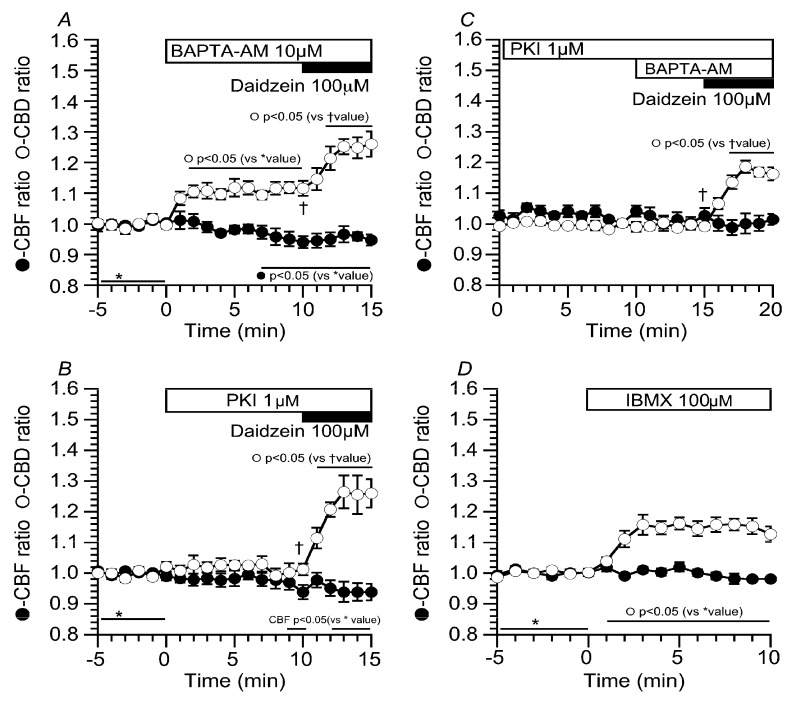
Effects of Ca^2+^ and cyclic adenosine monophosphate (cAMP) on daidzein actions. Experiments were carried out in the CO_2_/HCO_3_^−^-free solution. (**A**) BAPTA-AM (10 µM, *O*,*O*′-Bis(2-aminophenyl)ethyleneglycol-*N*,*N*,*N*′,*N*′-tetraacetic acid, tetraacetoxymethyl ester). To chelate intracellular Ca^2+^, cHNECs were treated with 10 µM of BAPTA-AM. The addition of BAPTA-AM increased CBD and gradually decreased CBF, suggesting that an extremely low [Ca^2+^]_i_ inhibits phosphodiesterase 1 (PDE1) to accumulate cAMP. Further stimulation with daidzein increased CBD, but not CBF. (**B**) PKI-A (1 µM, PKA inhibitor 14-22 amide). To inhibit PKA, cHNECs were treated with one µM of PKI-A. PKI-A did not affect CBD or CBF. Further daidzein stimulation increased CBD, but not CBF. (**C**) PKI and BAPTA-AM. Prior to BAPTA-AM treatment, cHNECs were treated with PKI-A for 10 min. The subsequent addition of BAPTA-AM (10 µM) did not increase CBD and CBF, suggesting that BAPTA-AM accumulates cAMP by inhibiting Ca^2+^-dependent PDE1. (**D**) 3-isobutyl-1-methylxanthine (IBMX) (100 µM). The addition of IBMX induced a small increase in CBD, but not CBF. The effects of cAMP accumulation on CBD and CBF in cHNECs were different from those in the lung airway ciliary cells of mice.

The results of this study indicate that daidzein stimulates Cl^−^ channels in cHNECs. We examined the effects of daidzein on intracellular Ca^2+^ concentration ([Ca^2+^]_i_) and cAMP accumulation in cHNECs. Experiments were carried out in the CO_2_/HCO_3_^−^-free solution. Prior to daidzein stimulation, cHNECs were treated with BAPTA-AM (10 µM, *O*,*O*′-Bis(2-aminophenyl)ethyleneglycol-*N*,*N*,*N*′,*N*′-tetraacetic acid, tetraacetoxymethyl ester) to inhibit [Ca^2+^]_i_ increase (Figure 9A). The addition of BAPTA-AM increased CBD, but not CBF. The further addition of daidzein increased CBD, but not CBF. Cells were also treated with PKI-A (1 µM, PKA inhibitor 14-22 amide) (Figure 9B). The addition of PKI-A alone did not change CBD and CBF, and then, the addition of daidzein increased CBD, but not CBF. Previous reports have already shown that airway ciliary cells have Ca^2+^-dependent phosphodiesterase 1 (PDE1) and BAPTA-AM (10 µM) stimulates cAMP accumulation by the inhibition of PDE1 [5,6,7]. We examined the effects of BAPTA-AM on CBD and CBF in the PKI-A-treated cHNECs (Figure 9C). The prior treatment of PKI-A abolished the CBD increase induced by BAPTA-AM, indicating that BAPTA-AM accumulates cAMP via Ca^2+^-dependent PDE1 inhibition in cHNECs. The further addition of daidzein still increased CBD, but not CBF. These results indicate that daidzein stimulates neither cAMP accumulation nor [Ca^2+^]_i_ increase in cHNECs. On the other hand, the addition of BAPTA-AM alone increased CBD, but not CBF. This CBD increase appears to be caused by cAMP accumulation via the inhibition of PDE1. However, cAMP accumulation has been shown to increase both CBA and CBF in mouse lung airway ciliary cells [4,5,6,7]. We examined the effects of cAMP accumulation on CBD and CBF in cHNECs. Cells were stimulated with 100 µM of IBMX. The addition of IBMX increased CBD by 20%, but not CBF. The extent of CBD increase stimulated by IBMX was smaller in cHNECs than in mouse lung airway ciliary cells [4,5,6,7].

### 2.6. Effects of Daidzein on CBD and CBF at 25 °C

**Figure 10:**
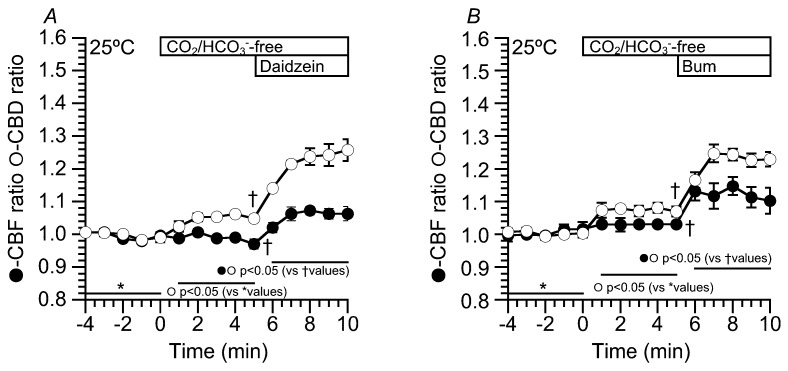
Effects of daidzein and bumetanide on CBD and CBF at 25 °C. Experiments were carried out under the CO_2_/HCO_3_^−^-free condition. (**A**) Daidzein (100 µM). Stimulation with daidzein increased both CBD and CBF. (**B**) Bumetanide (Bum, 20 µM). The addition of bumetanide also increased CBD and CBF.

In the previous studies, flavonoids, which stimulate Cl^−^ secretion, enhanced CBF in sinonasal ciliary cells [12,20,21,22]. Moreover, in the airway ciliary cells of rats, an isosmotic cell shrinkage, which decreases [Cl^−^]_i_, enhances CBF [23]. However, in this study, neither an [Cl^−^]_i_ decrease nor daidzein stimulation did not induce any CBF increase in cHNECs. The difference between previous studies and the present study is the temperature. The previous studies were carried out at room temperature or not completely controlled temperature, whereas the present study was carried out at 37 °C. The effects of daidzein (100 µM) and bumetanide (20 µM) on CBF and CBD were examined at 25 °C (Figure 10). The experimental protocol was similar to Figure 2 and Figure 6. The switch to CO_2_/HCO_3_^−^-free solution increased CBD, but not CBF. The values of CBD and CBF five min after the switch (Figure 10A) were 1.05 ± 0.01 (*n* = 4) and 0.97 ± 0.02 (*n* = 4). Further stimulation with daidzein (100 µM) increased both CBD and CBF. The values of CBD and CBF five min after daidzein addition were 1.26 ± 0.03 (*n* = 4) and 1.06 ± 0.02 (*n* = 4), respectively (Figure 10A). Thus, daidzein stimulation increased CBF at 25 °C. Moreover, bumetanide (20 µM) was used to decrease [Cl^−^]_i_. Just before the addition of bumetanide, the values of CBD and CBF were 1.07 ± 0.02 (*n* = 4) and 1.03 ± 0.02 (*n* = 4) (Figure 10B). The addition of bumetanide (20 µM) increased both CBD and CBF. The values of CBD and CBF five min after bumetanide addition were 1.23 ± 0.02 (*n* = 4) and 1.10 ± 0.03 (*n* = 4) (Figure 10B). Thus, an [Cl^−^]_i_ decrease enhanced both CBD and CBF at 25 °C and a CBF increase stimulated by a low [Cl^−^]_i_ value was dependent on the temperature.

## 3. Discussion

The present study demonstrated that daidzein enhances CBD, but not CBF, and accelerates the ciliary transport mediated via an [Cl^−^]_i_ decrease in primary culture cHNECs at 37 °C.

Previous studies have shown that daidzein stimulates a transient increase in NPPB-sensitive short circuit currents in renal A6 cells, indicating that daidzein stimulates Cl^−^ secretion [13]. This transient increase in the short circuit current was caused by an activation of Cl^−^ channels, not Na^+^/K^+^/2Cl^−^ cotransport (NKCC) [13,24]. In this study, daidzein stimulated a decrease in [Cl^−^]_i_, which was inhibited by NPPB. Daidzein enhanced the Cl^−^ efflux via Cl^−^ channels induced by a Cl^−^-free NO_3_^−^ solution. Moreover, under an extremely low [Cl^−^]_i_ condition, in which there is no driving force for Cl^−^ efflux via Cl^−^ channels, daidzein did not induce any decrease in [Cl^−^]_i_. The cHNECs have already been shown to express many types of Cl^−^ channels, such as CFTR (cystic fibrosis transmembrane conductance regulator) and TMEM16A (transmembrane member 16A) [21]. These observations indicate that daidzein activates Cl^−^ channels in cHNECs.

Daidzein increased CBD, which was mediated via a decrease in [Cl^−^]_i_, in cHNECs at 37 °C. Moreover, it increased both CBD and CBF at 25 °C. The ciliary beating is generated by two molecular motors, outer dynein arms (ODAs) and inner dynein arms (IDAs). ODAs control CBF, and IDAs control waveforms, including CBD [25,26]. Intracellular Cl^−^ appears to modulate both IDAs and ODAs in cHNECs. Decreases in [Cl^−^]_i_ have already been shown to modulate various cellular functions, such as Na-permeable channels, Ca^2+^-regulated exocytosis, and airway ciliary beating [11,19,23,27]. The mechanisms regulating ODA or IDA via intracellular Cl^−^ are unknown. A previous study showed that the microtubule activity in brain cytoplasmic dynein (ATPase activity) is enhanced by a low concentration of KCl, which increases dynein affinity to the microtubules [28]. Similar mechanisms may modulate CBD and CBF by regulating the ATPase activities of IDAs and ODAs. For example, IDAs or ODAs binding with Cl^−^ may reduce their ATPase activities in a temperature-dependent manner. Further studies are required to clarify the mechanisms.

The activation of Cl^−^ secretion has been shown to stimulate a CBF increase at 23 °C [20]. The activation of Cl^−^ secretion appears to decrease [Cl^−^]_i_ due to the activation of Cl^−^ efflux [11,24,29]. In the present study, an [Cl^−^]_i_ decrease enhanced CBD and CBF at 25 °C. These observations suggest that the activation of ciliary beating during the activation of the Cl^−^ secretion is caused by a [Cl^−^]_i_ decrease.

However, the present study exhibited that a decrease in [Cl^−^]_i_ increases CBD, but not CBF, in cHNECs at 37 °C. In the activation of the mucociliary clearance, CBF is thought to be a key factor [12,20,21,22], and was measured as an indicator of ciliary beating activity. Our previous study showed that an increase in ciliary bend amplitude, such as ciliary beat angle (CBA), also plays a crucial role for activating the mucociliary transport in the airways [4]. The present study demonstrated that an increase in CBD stimulates ciliary transport in cHNECs in primary culture. These observations indicate that CBD increase is also the key factor for stimulating mucociliary transport in the sinonasal epithelium.

The isoflavones, including daidzein, are compounds that are contained in many plant foods, such as soybeans, and are biologically active compounds that have crucial impacts on human health, such as anti-inflammatory, anti-allergic, and antioxidant actions. Their safety profile has already been established. Daidzein is composed of isoflavones, and is an inactive form of genistein, which is an inhibitor of protein tyrosine kinase. A previous study suggested that the structure of isoflavone, rather than flavone, plays a crucial role for activating Cl^−^ channels. Moreover, the presence of 5-hydroxy in isoflavone or flavone, such as in genistein or apigenin, stimulates Na^+^/K^+^/2Cl^−^ cotransporter; however, daidzein, an isoflavone with no 5-hydroxy, does not [13]. These observations may be important to design agents for activating Cl^−^ channels.

It is difficult to increase daidzein to its effective concentration in plasma, because orally-administered daidzein is minimally absorbed from the gut, and is metabolized to dihydrodaidzein by gut microflora, which binds to estrogen receptors and exerts antioxidative actions [30]. A previous study suggested that quercetin, a flavonoid, when added from the apical side stimulates CBF and CFTR in the human sinonasal epithelium [20]. Based on this observation, daidzein may stimulate Cl^−^ channels and CBD in nasal epithelia when administered to the nasal epithelium directly.

The present study suggests that the effects of cAMP accumulation in cHNECs are different from those in the ciliary cells of the trachea or lung airways. In lung airway ciliary cells, IBMX increased both CBD and CBF by 80% [5]. However, in cHNECs, IBMX increased CBD by only 20%, and did not increase CBF. We also observed a small CBF increase stimulated by IBMX in the nasal ciliary cells of mice [31]. The low extent of CBD increase, and no CBF increase, in response to cAMP accumulation is a characteristic feature of sinonasal ciliary cells, including cHNECs.

We used cHNECs from patients who had chronic sinusitis and allergic rhinitis. In both cases, daidzein stimulated an increase in CBD. This suggests that the action of daidzein on CBD is unlikely to be affected by these diseases. We also are aware that the primary culture condition changes the cellular characteristics affected by these diseases.

## 4. Materials and Methods

### 4.1. Ethical Approval

This study was approved by the ethics committees of the Kyoto Prefectural University of Medicine (RBMR-C-1249-4) and Ritsumeikan University (BKC-HM-2018-022), and informed consent was obtained from patients prior to surgery (No. RBMR-C-1249-2). Human nasal tissue samples (nasal polyp, uncinate process or inferior turbinate) were resected from patients requiring surgery for chronic sinusitis or allergic rhinitis. The samples were immediately cooled in the control solution (4 °C) and kept until cell isolation.

### 4.2. Solution and Chemicals

The CO_2_/HCO_3_^−^-containing control solution contained (in mM): NaCl, 121; KCl, 4.5; NaHCO_3_, 25; MgCl_2_, 1; CaCl_2_, 1.5; Na-HEPES, 5; H-HEPES, 5; and glucose, 5. To prepare the CO_2_/HCO_3_^−^-free control solution, NaHCO_3_ was replaced with NaCl, and to prepare the Cl^−^-free solution, Cl^−^ was replaced with NO_3_^−^. The CO_2_/HCO_3_^−^-containing solutions were aerated with 95% O_2_ and 5% CO_2_, and the CO_2_/HCO_3_^−^-free solutions were aerated with 100% O_2_. The pH of the solutions was adjusted to 7.4 by adding 1N-HCl or 1N-HNO_3_, as appropriate. The experiments were carried out at 37 °C. Heparin, elastase, bovine serum albumin (BSA), dimethylsulfoxide (DMSO), penicillin, streptomycin, trypsin, trypsin inhibitor, and daidzein were purchased from Wako Pure Chemical Industries, Ltd. (Osaka, Japan). DNase I, bumetanide, 5-Nitro-2-(3-phenylpropylamino)benzoic acid (NPPB), and amphotericin B were obtained from Sigma Chemical Co. (St Louis, MO, USA), and MQAE (*N*-ethoxycarbonylmethyl-6-methoxyquinolinium bromide) was obtained from Dojindo Laboratories. (Kumamoto, Japan). All of the reagents were dissolved in DMSO and prepared to their final concentrations immediately before the experiments. The DMSO concentration did not exceed 0.1%, and DMSO at this concentration had no effect on CBF, CBA, or CBD [4,5,6,7,23].

All culture medium was purchased from STEMCELL Technologies (Vancouver, BC, Canada) [32]. The complete PneumaCult-Ex medium contained PneumaCult-Ex 50 × Supplement (20 µL/mL), hydrocortisone (2.5 µL/mL), penicillin (100 unit/mL)m and streptomycin (100 µg/mL) in the PneumaCult-Ex Basal Medium, and the complete PneumaCult-ALI medium contained PneumaCult-ALI 10 × Supplement (0.1 mL/mL), PneumaCult-ALI Maintenance Supplement (10 µL/mL), heparin (1 µL/mL), hydrocortisone (2.5 µL/mL), penicillin (100 unit/mL), and streptomycin (100 µg/mL) in the PneumaCult-ALI Basal Medium.

### 4.3. Cell Preparation and Culture

The samples were treated with elastase to isolate ciliated human nasal epithelial cells (cHNECs) [4,5,6,7,8,16,23,33,34]. Briefly, the resected samples were incubated in the control solution containing elastase (0.02 mg/mL), DNase I (0.02 mg/mL), and BSA (3%) for 40 min at 37 °C. Following this incubation, the samples were minced by fine forceps in the control solution containing DNase I (0.02 mg/mL) and BSA (3%). The cells were washed three times by centrifugation (160× *g* for 5 min) and then suspended in the control solution. The cells were sterilized by amphotericin B (0.25 µg/mL) in Hams’s F-12 with L-Gln for 15 min. After centrifugation, cells were plated in collagen-coated flasks (Corning, 25 cm^2^) with complete PneumaCult-Ex medium. The complete PneumaCult-Ex medium was changed every second day until cells reached confluence [32]. After becoming confluent, the cells were isolated from the flask by trypsin treatment. Culture cells in the flask were washed with PBS (five mL), and then were incubated with 2 mL HBSS (Hanks’ balanced salt solution) containing 0.1 mM of EGTA (ethylene glycol tetraacetic acid) and 0.025% trypsin for 10 min at 37 °C. Following this incubation, HBSS (2 mL) containing a trypsin inhibitor (1 mg/mL) was added. The cell suspension was washed by centrifugation (160× *g* for five min). After washing, the cells were suspended in complete PneumaCult-Ex medium (6 mL), and the number of cells was counted. Next, cells (1–2 × 10^6^ cells/insert, 400 μL) were seeded on culture inserts (3470, 6.5-mm Transwell filter, Costar Corporation) and cultured in the insert, which was bathed in the complete PneumaCult-Ex medium (500 μL) in the basal chamber. When the cells reached confluence (four to six days), the medium was removed from the apical side, and the medium of the basolateral side was replaced with complete ALI (air liquid interface) medium (ALI condition). Cells were cultured under the ALI condition for three to four weeks, and they differentiated into cHNECs. These cHNECs were used for the experiments.

### 4.4. CBD and CBF Measurements

The permeable support filter with the cHNECs grown under the ALI condition was cut into small pieces (square, two to five mm per side). A piece of filter with cHNECs was placed on a coverslip that was precoated with Cell-Tak (Becton Dickinson Labware, Bedford, MA, USA). A coverslip was set into a microperfusion chamber (20 μL) that was mounted onto an inverted light microscope (ECLIPS Ti, NIKON, Tokyo, Japan) connected to a high-speed camera (FASTCAM-1024PCI, Photron Ltd., Tokyo, Japan). The stage of the microscope was heated and kept at 37 °C, because CBF depends on temperature [35,36]. The cells were perfused at 200 μL/min with the CO_2_/HCO_3_^−^-containing control solution aerated with a gas mixture (95% O_2_ and 5% CO_2_) at 37 °C. The video images of cHNECs were recorded for two seconds at 500 fps. Most of the beating cilia were viewed from the apical surface of the cHNEC cell sheet. When we set a line “a–b’’ on a beating cilium in the recorded video images (Figure 1A2), the image analysis program (DippMotion 2D, Ditect, Tokyo, Japan) reported the image showing the time course of changes in the light intensities along the line “a–b” (Figure 1C). The reported image shows the waveform of the beating cilium (Figure 1C). The peak of the waveform “s” shows the start of the effective stroke, and the bottom “e” shows the end of the effective stroke; the distance between the peak and bottom is the CBD (Figure 1C). The CBD and CBF ratios (CBD_t_/CBD_0_ and CBF_t_/CBF_0_), the values of which were normalized by the control values, were used to compare these parameters among experiments. CBDs or CBFs measured every one min during control perfusion (five min) were averaged, and the averaged value was used as CBD_0_ or CBF_0_. The subscript “0’’ or “t’’ indicates the time before or after the start of experiments, respectively. Each experiment was carried out using five to nine cover slips from three to six inserts. In each coverslip, we selected one to three cells and measured their CBDs and CBFs. The averaged CBD ratio and CBF ratio calculated from three to eight cells were plotted in the figures, and “n’’ shows the number of cells.

### 4.5. Measurement of [Cl^−^]_i_

Changes in [Cl^−^]_i_ were monitored using the fluorescence of MQAE (a chloride fluorescence dye) [16]. The cHNECs in culture were incubated with an EGTA solution for 10 min to isolate cHNECs from the filter. The isolated cHNECs were incubated with 10 mM MQAE for 45 min at 37 °C. MQAE was excited at 780 nm using a two-photon excitation laser system (MaiTai, Spectra-Physics), and the emission was 510 nm. The ratio of fluorescence intensity (F_0_/F_t_) was calculated as an index of [Cl^−^]_i_. The subscript “0’’ or “t’’ indicates the time before or after the start of experiments, respectively.

### 4.6. Observation of Latex Microbead Transport in cHNECs

To measure the rate of ciliary transport in cHNECs, we used latex microbeads, which were added on the apical surface of cHNECs [4]. The insert with the cHNECs was set into a culture dish whose bottom plate was made of a cover slip, and bathed in the HCO_3_^−^-free solutions (1 mL). The culture dish was set on the stage of an inverted light microscope (ECLIPS Ti, NIKON, Tokyo, Japan) connected to a high-speed camera (FASTCAM-1024PCI, Photron Ltd., Tokyo, Japan). The microscope stage was heated to 37 °C. The HCO_3_^−^-free solution (20 μL, 37 °C) containing microbeads (one μm diameter, polystyrene microspheres, Polysciences Inc., Warrington, PA, USA), was added into the apical side of the cHNECs. The movement of the latex microbeads, driven by the beating cilia of the cHNECs, was recorded for 3.2 s at 60 fps by a high-speed camera. Upon the addition of the microbeads on the apical surface of cHNECs, some microbeads reached the surface of cHNECs and were transported according to the surface flow driven by the ciliary beating. We selected a cHNEC on the filter and observed its microbead movements.

### 4.7. Statistical Analysis

Data are expressed as the means ± SEM. Statistical significance was assessed by the analysis of variance (ANOVA), Student’s paired t-test, or Student’s unpaired *t*-test, as appropriate. Differences were considered significant at *p* < 0.05.

## Supplementary Materials

Supplementary materials can be found at http://www.mdpi.com/1422-0067/19/12/3754/s1. **S1**. Supplementary videos of beating cilia of cHNECs (apical view) (A) Before addition of daidzein. (B) 15 min after daidzein stimulation. Daidzein enhanced the amplitude of ciliary beating. **S2**. Supplementary videos of the microbead movement before (A) and five min after daidzein stimulation (B). Daidzein enhanced the micro-beads movements.
